# A Sensorless Predictive Current Controlled Boost Converter by Using an EKF with Load Variation Effect Elimination Function

**DOI:** 10.3390/s150509986

**Published:** 2015-04-28

**Authors:** Qiaoling Tong, Chen Chen, Qiao Zhang, Xuecheng Zou

**Affiliations:** 1 School of Optical and Electronic Information, Huazhong University of Science and Technology, Wuhan 430074, China; E-Mails: tongqiaoling@hust.edu.cn (Q.T.); hust_chenchen@163.com (C.C.); xuechengzou@163.com (X.Z.); 2 IMRA Europe S.A.S., Brighton BN19RS, UK

**Keywords:** boost converter, sensorless predictive current control, extended Kalman filter, load variation effect elimination

## Abstract

To realize accurate current control for a boost converter, a precise measurement of the inductor current is required to achieve high resolution current regulating. Current sensors are widely used to measure the inductor current. However, the current sensors and their processing circuits significantly contribute extra hardware cost, delay and noise to the system. They can also harm the system reliability. Therefore, current sensorless control techniques can bring cost effective and reliable solutions for various boost converter applications. According to the derived accurate model, which contains a number of parasitics, the boost converter is a nonlinear system. An Extended Kalman Filter (EKF) is proposed for inductor current estimation and output voltage filtering. With this approach, the system can have the same advantages as sensored current control mode. To implement EKF, the load value is necessary. However, the load may vary from time to time. This can lead to errors of current estimation and filtered output voltage. To solve this issue, a load variation elimination effect elimination (LVEE) module is added. In addition, a predictive average current controller is used to regulate the current. Compared with conventional voltage controlled system, the transient response is greatly improved since it only takes two switching cycles for the current to reach its reference. Finally, experimental results are presented to verify the stable operation and output tracking capability for large-signal transients of the proposed algorithm.

## 1. Introduction

In recent years the current mode digital controlled DC-DC converter has become a hot research topic [1,2,3,4,5,6,7]. As one of the most used DC-DC converters, the research on boost converters control has been well developed [8,9,10]. Compared with voltage mode controlled system, it has higher response speed and larger loop gain bandwidth. However, to realize high quality current feedback control, precision current sensors are essential. State-of-art current sensing technologies are reviewed in [11]. There are many kinds of technologies for current sensing, for example the giant magnetoresistance effect based current sensor provides low cost isolation solution [12,13]. However, for a boost converter, there are three most common types of current sensors. The first type uses a shunt resistor in series with the switching device, the second type uses current mirror to reconstruct the switch component current [14,15], and the third type uses Hall effect sensors [16]. The first type may add power losses while the second type may suffer EMI problem [17]. The third type is the most accurate method, and it can be designed highly immune to EMI [18]. However, the cost of most Hall current sensors is relatively high. The current sensors and their signal processing circuits introduce delay and noise to the control circuitry and also contribute to the overall cost of the converter. Therefore, the sensorless current controlled boost converter which acts in current control mode with all the above advantages but without needing a current detecting module has got great potentials in both academic and industrial applications.

To realize sensorless current control, a current observer is normally used to estimate the current. Performance of the current observer deeply relies on the accuracy of system modeling [19,20,21]. In [22,23,24], a variety of boost converter modeling strategies are investigated. P. Midya proposed a sensorless current control strategy based on current observer in 2001 [25], the model is quite accurate. However, for real time digital control, the implementation of this strategy is far too complex. An easier algorithm by feed forward current observer based on the input voltage was published in 2004 [26], the input voltage feed forward was introduced in the observer, and it can effectively avoid the impact of the output voltage variations on the current observer. In this algorithm, however, the influence of the parasitic parameters was not considered and the current estimation error is relatively large. To improve the boost converter dynamic response, a Control-Lyapunov Function based sensorless current control strategy for a boost PFC is proposed in [27]. A new sensing technique by measuring the maximum and minimum values to get the output voltage mean value is proposed, which is able to eliminate the double frequency ripple. Thus, the bandwidth of the voltage controller can be increased significantly. Cho investigated a state observer based sensorless controller using Lyapunov’s direct method for boost converters [28]. A state observer is constructed to estimate the inductor current through input and output voltages together with a switch control signal. The system shows good performance in terms of transient response. 

An optimized reduced order current observer is proposed for a buck converter by Min [1]. Valley current control with trailing edge (TE) PMW modulation is employed. The current estimation is quite accurate and the algorithm is easy to implement. In [29], a reduced order current observer is used for current estimation for a boost converter. Its current control mode is different from [1] since the peak current control with TE PWM modulation is applied. According to [30], this kind of combination can cause the system unstable. To solve this issue, the reference valley current of two switching cycle ahead is derived from the reference peak value, and then the duty ratio of next switching cycle is calculated through this reference valley current. With this approach, the stable peak current control is realized. Furthermore, in some applications, if the estimated current is average current, the average current control can be implemented directly to reduce the computational complexity. Moreover, the main contribution for this literature is that the root reason for output steady state error is found out. To eliminate the voltage steady state error and achieve high accuracy current estimation, a comprehensive compensation strategy was proposed to eliminate the effect of component parasitic parameters and signal sampling error. 

To choose a current control algorithm, predictive current control (PCC) is a good candidate. It is feathered with high robustness, high response speed and low implementation complexity. Therefore, combining the sensorless current control with PCC is an optimized strategy for boost converter control. There are many literatures focusing on PCC. In [31], Stephane Bibian proposed a high performance predictive Dead-beat digital control algorithm to eliminate the computational delay affection. Since the duty ratio is updated every two switching cycles, its response speed is not high. To achieve high response speed, Chen proposed an algorithm to eliminate the inductor current disturbance in two switching cycles for peak, average and valley current control modes [30]. Lai further investigated PCC based peak current mode control in [32]. The effectiveness to eliminate the disturbance in limit cycles by PCC with leading edge PWM modulation scheme was verified by theoretical derivation. In [33], the authors combined the predictive and feed forward control with the PID controller to achieve fast transient response and low overshoot. Its transient response time is reduced by approximately 50%.

The aforementioned literature has made huge contributions to the development of boost converter control. In this paper, an accurate boost converter model, which includes a number of parasitics, is derived. As can been seen from this motel, the boost converter is a nonlinear system. Since EKF is suitable for nonlinear system state observation and measurement noise filtering, so it is chosen to act as a current sensor to estimate the boost converter inductor current. There is much literature on EKF implementation on state estimation of nonlinear systems [34,35]. However, for a boost converter, the load value is necessary for EKF design and it is subject to change with working conditions. This variation can lead to errors of current estimation as well as output voltage filtering. Unfortunately, there has not been any solution for the load variation issue in EKF based current observer yet. Therefore, a load variation effect elimination (LVEE) method is proposed together with the EKF. The current estimation accuracy, system dynamic response and no output voltage steady state error can be guaranteed by the introducing of LVEE module. What is more, the implementation of a PCC controller improves the system dynamic performance. The proposed method can be used in applications with mainly resistive load such as resistive electrical heating, electric oven, filament lamp, *etc.* For inductive and capacitive load such boost converter in Hybrid Electric Vehicles, inductive oven and battery charger, LVEE module needs further investigation and this is for the next stage research. However, for practical applications, more elements should be taken into account. First, it is suitable for CCM condition. Extra modifications are needed if the application works in DCM condition. Second, the temperature variation and aging effect can cause the system parasitics subject to change. Especially inductor parasitic resistance is easily affected by temperature and capacitor ESR changes dramatically (100% increase) through aging effect. If the system model is not updated accordingly, it can lead to current estimation error. A look up table can be used to store these parameters under different conditions and the model parameters can be updated. However, the ultimate way to solve this issue is to use online parameters identification. Finally, please bear in mind, there is no pure resister in the real world, the load should be treated as a parasitic inductor in series with a resistor or inductive capacitive types with resistive parasitic, which depends on the actual applications. In this paper, the load is treated as a resistor because most academic literatures use this way to make the presentation easy to understand. In addition, the load parasitic inductance for the test in this paper is negligible.

The paper is organized as follows. In Section 2, the overall control structure and mathematical of a boost converter with proposed algorithm is presented. An accurate model of this boost converter, which contains a number of parasitics, is also derived. In Section 3, the current estimation module, which consists of an EKF together with a LVEE module, is designed. It can not only estimate the inductor average current accurately and filter the measurement noise of output voltage but also can be helpful for improving system steady and dynamic performance. In addition, the detailed analysis on LVEE module is carried out to explain its effect on eliminating output voltage steady state error. An average current mode based PCC controller is designed in Section 4. The error between reference current and estimated average current can be eliminated in two switching cycles. Finally, the experimental results are given in Section 5.

## 2. System Control Structure and Mathematical Model

### 2.1. The System Control Structure

**Figure 1:**
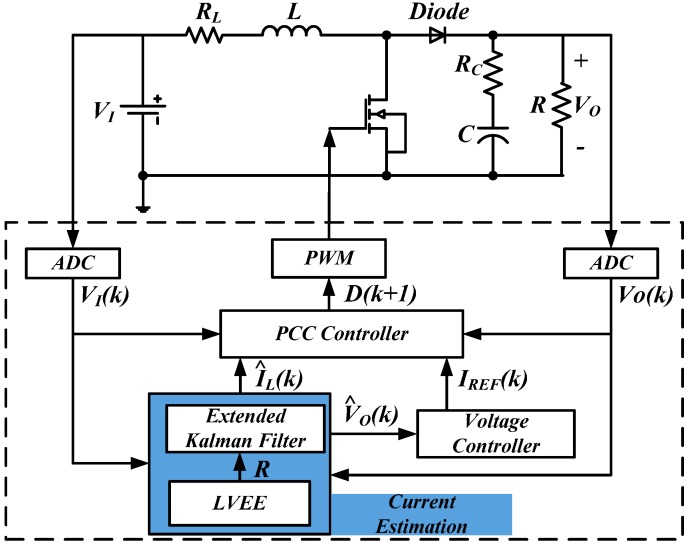
Control diagram of an EKF based sensorless current controlled boot converter.

The structure of a boost converter with proposed algorithm is shown in Figure 1. The system is comprised of two control loops. The outer loop is a voltage control loop, using a PI controller to regulate the output voltage ${\hat{V}}_{O}\left(k\right)$, which is also filtered by the EKF. Its output is the current reference. The inner loop is a current control loop consists of a current estimation module and a PCC controller. The whole process for current loop design is investigated in this paper.

The current estimation module consists of an EKF and a LVEE module. The inductor average current estimation and output voltage measurement noise filtering are realized by the EKF. Load variations are eliminated by the LVEE. For current control, the average current control mode is used. The PWM duty ratio of next switching cycle is derived according to the sampled input and output voltages. Then the error between reference and actual average currents can be eliminated.

### 2.2. The Accurate Mathematical Model of the Boost Converter

Since the model accuracy affects the performance of current estimation, an accurate system model is necessary. An accurate model with a series of parasitic parameters for a boost converter is derived as follows.

**Figure 2:**
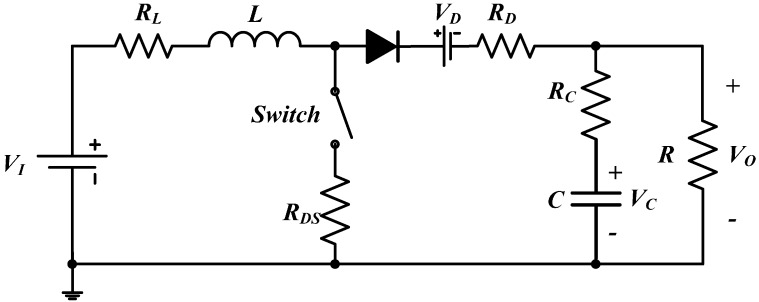
The accurate model of a boost converter with a number of parasitic parameters.

Figure 2 shows the equivalent model of a boost converter, a series of parasitic parameters are included. *R_L_*, *R_DS_*, *R_D_*, *V_D_* and *R_C_* are parasitic resistor of inductor, switching on resistor of MOSFET, conduction resistor of diode, forward voltage drop of diode, and equivalent series resistor (ESR) of capacitor, respectively.

Setting inductor current *I_L_*(*t*) and capacitor voltage *V_C_*(*t*) as the state variables, the system state function is derived as follows. 

When the switch is on, the capacitor is discharged to supply energy to load, then

(1) $$V_{O}\left(t\right)=V_{C}\left(t\right)-\frac{V_{O}\left(t\right)}{R}R_{C}$$

Equally as Equation (1)

(2) $$V_{O}\left(t\right)=\frac{R}{R+R_{C}}V_{C}\left(t\right)$$

During switching on period, discharging current of capacitor is −*V_O_*(*t*)/*R*, the system state function is

(3) $$\begin{array}{l} L\frac{dI_{L}\left(t\right)}{dt}=V_{I}-I_{L}\left(t\right)\left(R_{L}+R_{DS}\right)\\ C\frac{dV_{C}\left(t\right)}{dt}=-\frac{V_{C}\left(t\right)}{R+R_{C}} \end{array}$$

When the switch is off, the inductor charges capacitor and provides energy for load

(4) $$V_{O}\left(t\right)=V_{C}\left(t\right)\text{+}\left[I_{L}\left(t\right)-\frac{V_{O}\left(t\right)}{R}\right]R_{C}$$

Equally as Equation (4)

(5) $$V_{O}\left(t\right)=\frac{R}{R+R_{C}}\left[V_{C}\left(t\right)\text{+}I_{L}\left(t\right)R_{C}\right]$$

During the switching off period, charging current of capacitor is *I_L_*(*t*) − *V_O_*(*t*)/*R*, the system state function is

(6) $$\begin{array}{l} L\frac{dI_{L}\left(t\right)}{dt}=V_{I}-V_{D}-\left(R_{L}\text{+}R_{D}+\frac{RR_{C}}{R+R_{C}}\right)I_{L}\left(t\right)-\frac{R}{R+R_{C}}V_{C}\left(t\right)\\ C\frac{dV_{C}\left(t\right)}{dt}=\frac{R}{R+R_{C}}I_{L}\left(t\right)-\frac{V_{C}\left(t\right)}{R+R_{C}} \end{array}$$

Setting $X\left(t\right)={\left[\begin{array}{cc} X_{1}\left(t\right) & X_{2}(t) \end{array}\right]}^{T}={\left[\begin{array}{cc} I_{L}\left(t\right) & V_{C}(t) \end{array}\right]}^{T}$, Equations (3) and (6) can be presented as

(7) $$\text{Swithching on}:\;\dot{X}\left(t\right)=F_{1}X\left(t\right)+G_{1}$$

(8) $$\text{Swithching off}:\;\dot{X}\left(t\right)=F_{2}X\left(t\right)+G_{2}$$

where *F*_1_, *G*_1_, *F*_2_ and *G*_2_ are

(9) $$F_{1}=\left[\begin{array}{cc} -\frac{R_{L}+R_{DS}}{L} & 0\\ 0 & -\frac{1}{C\left(R+R_{C}\right)} \end{array}\right]\;G_{1}=\left[\begin{array}{c} \frac{V_{I}}{L}\\ 0 \end{array}\right]$$

(10) $$F_{2}=\left[\begin{array}{cc} -\frac{RR_{C}+\left(R+R_{C}\right)\left(R_{L}+R_{D}\right)}{L\left(R+R_{C}\right)} & -\frac{R}{L\left(R+R_{C}\right)}\\ \frac{R}{C\left(R+R_{C}\right)} & -\frac{1}{C\left(R+R_{C}\right)} \end{array}\right]\;G_{2}=\left[\begin{array}{c} \frac{V_{I}-V_{D}}{L}\\ 0 \end{array}\right]$$

At first, integrate Equations (7) and (8), respectively, during switching on 0 ~ *dT* and switching off *dT* ~ *T* periods, then add them together and divide the sum by switching period *T*, the average state function during the whole switching cycle is obtained as Equation (11)

(11) $$\dot{X}\left(t\right)=\left[F_{2}+\left(F_{1}-F_{2}\right)d\left(t\right)\right]X\left(t\right)+\left(G_{1}-G_{2}\right)d\left(t\right)+G_{2}$$

According to capacitor charging balance principle, when the system is in steady state, average output voltage *V_O_* is equal to average capacitor voltage *V_C_*. So *X*(*t*) can be described as $X\left(t\right)={\left[\begin{array}{cc} I_{L}\left(t\right) & V_{O}(t) \end{array}\right]}^{T}$.

There is a nonlinear item *X*(*t*)*d*(*t*) in Equation (11), which demonstrates that boost converter is a nonlinear system. So the EKF, which is always used for nonlinear systems, is chosen for current estimation and output voltage filter.

## 3. Proposed Current Estimation Strategy

In this section, an EKF for boost converter current sensorless control is first proposed. Then a LVEE method is investigated. In addition, further theoretical analysis on the filtered output voltage steady state error elimination by LVEE module is carried out.

### 3.1. An EKF for Current Estimation

The accurate model of a boost converter has been built in Section 2. The EKF for current estimation and voltage filter can be derived accordingly. In order to realize the EKF based digital control, the state function of boost converter has to be converted to discrete domain at first.

Converting Equation (11) to discrete domain, a nonlinear random differential function of boost converter is obtained

(12) $$\begin{array}{l} X\left(k\right)=AX(k-1)+BX(k-1)d\left(k\right)+Cd\left(k\right)+D+w\left(k-1\right)\\ \;\;\;\;\;\;\;\;=f\left[X(k-1),d\left(k\right),w\left(k-1\right)\right] \end{array}$$

Measurement function is

(13) Z(k)=HX(k)+v(k)

where, the input is duty ratio *d*(*k*), *Z(k)* is the measurement variable, which is the output voltage; *V_O_*(*k*), *w*(*k* − 1) and *v*(*k*) are noises of the process and measurement. They are not coupled. *A*, *B*, *C* and *D* in discrete domain can be described as *A* = *I* + *TF*_2_, *B* = *T*(*F*_1_ − *F*_2_), *C* = *T*(*G*_1_ − *G*_2_), and *D* = *TG*_2_. *H* is the observation matrix in discrete domain and *H* = [1 0].

Essentially, an EKF consists of a group of mathematical functions to realize prediction, adjusts and estimation. By using EKF, the covariance of estimation error can be reduced as low as possible. According to Equation (12), the updating functions of EKF are

(14) $$\tilde{X}\left(k\right)=A\hat{X}(k-1)+B\hat{X}(k-1)d\left(k\right)+Cd\left(k\right)+D$$

(15) $$\tilde{P}\left(k\right)=A(k)P\left(k-1\right)A^{T}(k)+Q$$

Equations (14) and (15) are the state and covariance prediction equations. *Q* is a covariance. The state and covariance of current switching cycle can be derived from last switching cycle. Where $\tilde{X}\left(k\right)$ is the previous state estimation of the *k*th cycle, while $\hat{X}(k-1)$ is the post state estimation of the (*k* − 1)th cycle. $\tilde{P}\left(k\right)$ is the previous estimation covariance of the *kth* cycle, and *P*(*k* − 1) is the post estimation covariance of the (*k* − 1)th cycle. *A*(*k*) is a Jacob matrix of the process and it can be derived from Equation (16).

(16) $$A(k)=\frac{\partial f\left[X(k-1),d\left(k\right)\right]}{\partial X\left(k-1\right)}=A+Bd\left(k\right)$$

Using measurements to correct the previous state and covariance of estimation errors, then the measurement update matrix of EKF are

(17) $$Kg(k)=\tilde{P}(k)H^{T}{\left[H\tilde{P}(k)H^{T}+R\right]}^{-1}$$

(18) $$\hat{X}\left(k\right)=\tilde{X}(k)+Kg\left(k\right)\left[Z\left(k\right)-H\tilde{X}(k)\right]$$

(19) $$P(k)=[I-Kg(k)H]\tilde{P}(k)$$

Equations (17)–(19) are the correction equations for state and covariance predictions. *Kg*(*k*) is the filter gain. *R* is a covariance. All the equations of the EKF are presented from Equations (14) to (19). As above process shows, only input and output voltages need to be sampled. Then the inductor current can be estimated and the output voltage can be filtered.

### 3.2. The LVEE Module

Load*R* is involved both in *F*_1_ and *F*_2_. In practical, load varies with environmental and working conditions. If this variation is not considered, it can lead errors of current estimation and steady state output voltage. In this paper, the load *R* is replaced by an incremental resistance to eliminate the load variation effect. Therefore, the LVEE module is proposed.

Using average state method, in steady state, the relationship between the load current and inductor current *I_L_*(*k*) is described as Equation (20)

(20) $$I_{O}(k)=I_{L}(k)[1-d(k)]$$

The relationship between the output voltage and load current is

(21) $$V_{O}(k)=I_{O}(k)R$$

Combine Equations (20) and (21), the relationship between the inductor current and output voltage is obtained.

(22) $$R=\frac{V_{O}(k)}{I_{L}(k)[1-d(k)]}$$

Using outputs of EKF ${\hat{I}}_{L}(k)$ and ${\hat{V}}_{O}(k)$ to replace the actual output voltage *V_O_*(*k*) and inductor current *I_L_*(*k*) in Equation (22), then *R* is derived as the following incremental resistance.

(23) $$R=\frac{{\hat{V}}_{O}(k)}{{\hat{I}}_{L}(k)[1-d(k)]}$$

Substituting Equation (23) into *F*_1_ and *F*_2_, the load variation affection on EKF steady state outputs can be eliminated. The accuracies of current estimation and voltage regulation are improved. 

### 3.3. Analysis on the LVEE Module

To find out the effect of LVEE module on output voltage stead state error, theoretical analysis is carried out as following. *R_L_* is considered in the verification process. The state function of output voltage is derived from discretization of Equation (11).

(24) $$C\frac{{\hat{V}}_{O}(k+1)-V_{O}(k)}{T}=I_{L}(k)[1-d(k)]-\frac{V_{O}(k)}{R}$$

In steady state, the sampled output voltage stays constant, then *V_O_*(*k*) = *V_O_*(*k* + 1). Since steady state error of output voltage is $\Delta V_{O}(k)={\hat{V}}_{O}(k)-V_{O}(k)$, substituting Equation (23) into Equation (24), then

(25) $$\Delta V_{O}(k+1)=\frac{D^{\prime }(k)T}{C{\hat{V}}_{O}(k)}\left\{I_{L}(k)\Delta V_{O}(k)\text{+}V_{O}(k)\left[I_{L}(k)-{\hat{I}}_{L}(k)\right]\right\}$$

where *d'*(*k*) = 1 − *d*(*k*).

In steady state $\dot{X}\left(t\right)\text{=}0$, converting Equation (11) into discrete domain, *I_L_*(*k*) in steady state is described as

(26) $$I_{L}(k)\text{=}\frac{V_{I}(k)-d^{\prime }(k)V_{O}(k)}{R_{L}}$$

Estimated inductor current ${\hat{I}}_{L}(k)$ is expressed as Equation (27)

(27) $${\hat{I}}_{L}(k)\text{=}\frac{V_{I}(k)-d^{\prime }(k){\hat{V}}_{O}(k)}{R_{L}}$$

Subtracting Equation (26) with Equation (27), estimation error of inductor current is

(28) $$I_{L}(k)-{\hat{I}}_{L}(k)\text{=}\frac{d^{\prime }(k)\Delta V_{O}(k)}{R_{L}}$$

Substituting Equation (28) into Equation (25), then

(29) $$\frac{\Delta V_{O}(k+1)}{\Delta V_{O}(k)}=\frac{d^{\prime }(k)T}{C{\hat{V}}_{O}(k)}\left[I_{L}(k)\text{+}\frac{d^{\prime }(k)V_{O}(k)}{R_{L}}\right]$$

During *d*(*k*)*T* period, discharging current is *I_L_*(*t*)[1 − *d*(*k*)], the peak to peak value of output voltage *V_PP_* (voltage ripple) is shown as Equation (30)

(30) $$V_{PP}=\frac{\int_{0}^{d(k)T}I_{L}(t)[1-d(k)]dt}{C}=\frac{I_{L}(k)d(k)[1-d(k)]T}{C}$$

Substituting Equation (30) into Equation (29),

(31) $$\frac{\Delta V_{O}(k+1)}{\Delta V_{O}(k)}=\frac{V_{PP}}{d(k){\hat{V}}_{O}(k)}\text{+}\frac{T{d^{\prime }}^{2}(k)}{CR_{L}}\frac{V_{O}(k)}{{\hat{V}}_{O}(k)}\text{=}E_{1}+E_{2}$$

In practical, *V_PP_* is always far low than output voltage, which means *E*_1_ << 1. Normally *Td'*^2^(*CR_L_*) << 1, since the error between the input and output of EKF is low ${\hat{V}}_{O}(k)\approx V_{O}(k)$, then *E*_2_ << 1. So Equation (31) is less than 1, the error of EKF output decreases and finally equals to actual output voltage.

## 4. Average Current Control Based on PCC

In this paper, a leading edge PWM modulation scheme is used. According to [31], sub harmonic oscillation exists in average control mode. Because when the average current equals to reference current in steady state, the peak current may not be a constant value and this causes the oscillation. In this section, a novel PCC based control algorithm is proposed. When any disturbance happens in current control loop, the current controller regulates the peak current to constant at first, and then the average current is also regulated to reference value in the following switching cycles. 

**Figure 3:**
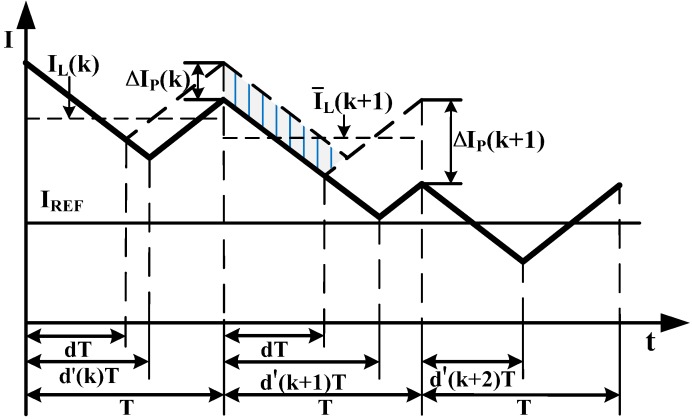
Inductor current waveform under proposed average current control mode.

Figure 3 shows the inductor current waveform under proposed average current control mode by using the leading edge PWM modulation method.

Assuming there is a disturbance on inductor peak current in the *k*th switching cycle, and it is described as Equation (32).

(32) $$\Delta I_{P}\left(k\right)=\left[M_{1}\left(k\right)\text{+}M_{2}\left(k\right)\right]\Delta d\left(k\right)T$$

where ∆*d*(*k*) = *d* − *d*(*k*), *d* is the duty ratio of steady state, and it can be expressed as *d* = *M*_2_(*k*)/[*M*_1_(*k*) + *M*_2_(*k*)]. *M*_1_(*k*) is the positive slope of inductor current in the *k*th switching cycle, and *M*_2_(*k*) is the negative slope absolute value. They are described as Equations (33) and (34).

(33) $$M_{1}\left(k\right)=\frac{V_{I}\left(k\right)-I_{AV}\left(k\right)\left(R_{L}+R_{DS}\right)}{L}$$

(34) $$M_{2}\left(k\right)=\frac{V_{O}\left(k\right)-V_{I}\left(k\right)+V_{D}+I_{AV}\left(k\right)\left(R_{L}+R_{D}+R_{COMP}\right)}{L}$$

where *R_COMP_* = *R_C_* + *d'd*/2*fC*, f is the switching frequency.

In Figure 3, the shade area is the difference required to maintain the (*k* + 1)th cycle peak current stays the same as the kth cycle and it can be derived as

(35) $$\Delta {\bar{I}}_{L}\left(k+1\right)=\frac{1}{2}\Delta I_{P}\left(k\right)\left[d\left(k\right)+d\right]$$

When the peak current of the (*k* + 1)th cycle stays constant, its average current is

(36) $${\bar{I}}_{L}\left(k+1\right)=I_{L}\left(k\right)-\Delta {\bar{I}}_{L}\left(k+1\right)$$

First, average current of the (*k* + 2)th is guaranteed equal to reference current by adjusting the duty ratio of the (*k* + 1)th cycle, and this duty ratio is derived from Equation (32).

(37) $$d\left(k\text{+}1\right)\text{=}d\text{+}\frac{\Delta I_{P}\left(k\text{+}1\right)}{\left[M_{1}\left(k+1\right)\text{+}M_{2}\left(k+1\right)\right]T}$$

As can be seen from Figure 3, if the errors of average and peak currents of the (*k* + 2)th cycle are all zero, peak current variation of the (*k* + 1)th cycle is

(38) $$\Delta I_{P}\left(k+1\right)=I_{REF}\left(k+1\right)-{\bar{I}}_{L}\left(k+1\right)$$

Because the switching period is relatively short compared with system electrical time constant, the slopes of the continuous two switching cycles can be regarded as constant *M*_1_(*k*) ≈ *M*_1_(*k* + 1), *M*_2_(*k*) ≈ *M*_2_(*k* + 1). Substituting Equation (38) into Equation (34), duty ratio of the (*k* + 1)th cycle is derived

(39) $$d\left(k+1\right)=d\text{+}\frac{I_{REF}\left(k+1\right)-{\bar{I}}_{L}\left(k+1\right)}{\left[M_{1}\left(k\right)+M_{2}\left(k\right)\right]T}$$

Using Equation (39) to regulate the system, ${\bar{I}}_{L}\left(k+2\right)$ in the (*k* + 2)th cycle is equal to reference current. Then proper duty ratio of the (*k* + 2)th cycle *d*(*k* + 2) is derived from Equation (39). *d*(*k* + 2) makes estimated average current equal to reference current and keeps peak current constant ∆*I_P_*(*k* + 2) = 0. So the proposed current control algorithm can eliminate the current in two switching cycles without causing any oscillations even *d* ≥ 0.5.

## 5. Experimental Results

In order to verify the proposed algorithm, a series of experiments in steady state and transient state with load and line voltage changing conditions are carried out for a boost converter. For comparison the same experiments are implemented by using conventional voltage control mode with the same hardware. Design parameters of the target boost converter are shown in Table 1.

**Table 1:** Specifications of the tested boost converter.

Input voltage	6 V
Output voltage	12 V
Rated output current (ROC)	0.5 A
Voltage ripple under ROC	1%
Switching frequency	50 kHz

The built boost converter consists of control and power sections. The core of the control section is a Texas Instruments digital signal processor (DSP) TMS320F2812. The power section includes the main power stage and signal sampling circuits and the input and output voltages are sampled at the beginning of each switching cycle. The switching device of the power stage is an Infineon BSZ110N06NS3 MOSFET, the output capacitor is Panasonic EEHZC1E101XP, and the diode is Liteon SB350. The components specifications are presented in Table 2.

For monitoring, the estimated average current is output synchronously by a 12-bit (digital to analog converter) DAC TVL5616. The actual inductor current is measured by a current probe with a resolution of 200 mV/A. For easy comparison, the DAC output is set at the same scale. In the following voltage waveforms Channel 1 is sampled by AC mode with fine resolution to show voltage ripple clearly while Channel 2 shows output voltage itself with the resolution of 2 v/div. In current waveforms, Channel 1 is the actual current waveform marked as *i_l_* and Channel 2 is the estimated average current waveform marked as *I_L_*.

**Table 2:** Specifications of hardware platform.

Inductance of the power inductor	120 µH
Inductor winding resistance	250 mΩ
Capacitance of the output capacitor	75 µF
ESR value of the output capacitor	50 mΩ
MOSFET R_DS_	11 mΩ
Diode forward Voltage	0.7 V
Diode forward resistance	100 mΩ

(1) Experiments for the LVEE module function verification

**Figure 4:**
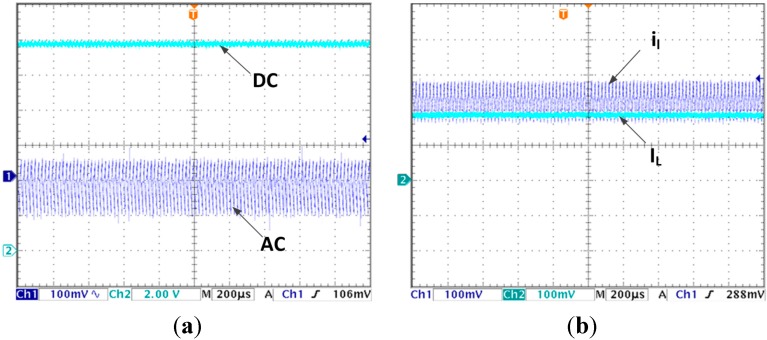
(**a**) Steady state output voltage without using LVEE module; (**b**) Steady state inductor current without using LVEE Module

Figure 4a,b are the steady state voltage and current waveforms, respectively, without using LVEE module. As shown in Figure 4a, the output voltage in steady state is 11.57 V and there is a steady state error up to 0.43 V. In Figure 4b, the estimated current average is 1.03 A while the actual average current should be 1.16 A, the current estimation steady state error is 0.13 A. 

**Figure 5:**
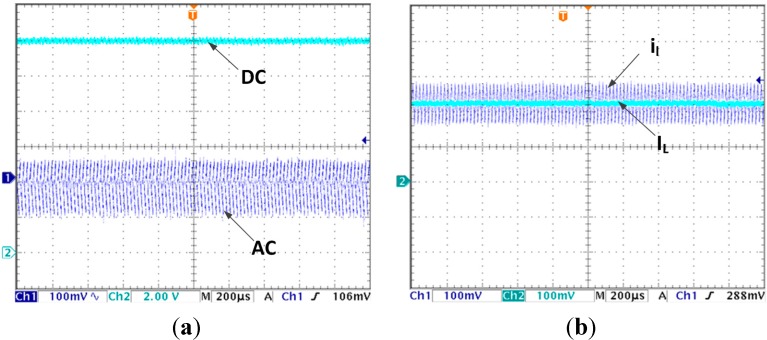
(**a**) Steady state output voltage by using LVEE module; (**b**) Steady state inductor current by using LVEE Module.

When the LVEE module is added, the steady state output voltage and current waveforms are presented in Figure 5a,b. Neither the output voltage nor the estimated average current has steady state error.

(2) Experiments with load changing condition

**Figure 6:**
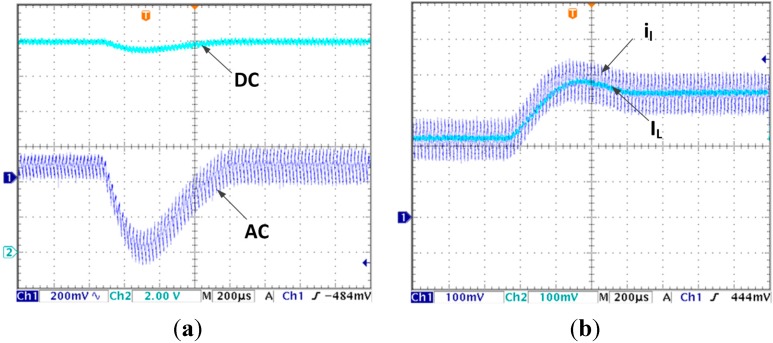
(**a**) Output voltage transient response due to load changes from 24 Ω to 16 Ω with proposed algorithm; (**b**) Inductor current transient response due to load changes from 24 Ω to 16 Ω with proposed algorithm

Figure 6a,b are the output voltage and inductor currents waveforms in load changing condition (R changes from 24 Ω to 16 Ω) by using the proposed algorithm. In Figure 6a, the output voltage declines to 11.52 V, then returns to 12 V within 710 µs after the load changes. As shown in Figure 6b, the actual inductor average jumps from 1.15 A to 1.77 A in 710 µs after the load changes. It equals to its estimated value.

**Figure 7:**
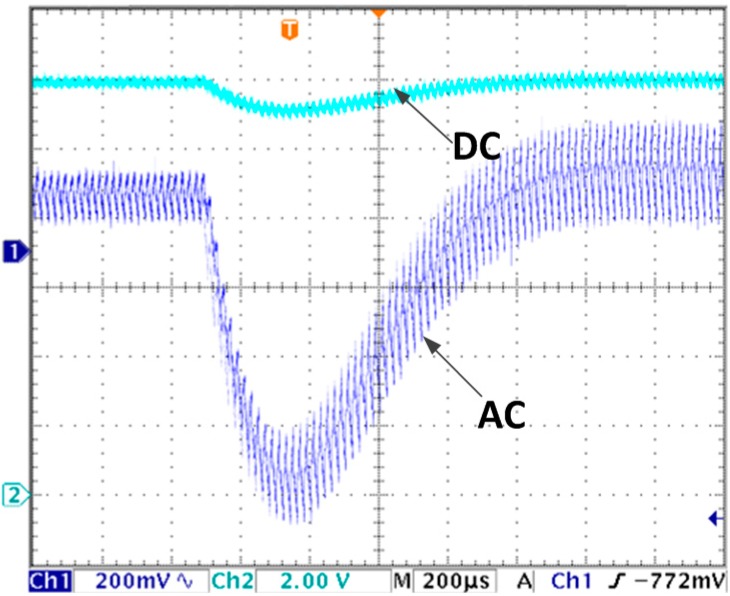
Output voltage transient response due to load changes from 24 Ω to 16 Ω with voltage control mode.

The output voltage waveform under load changing condition with conventional voltage control is shown as Figure 7. It decreases to 11.15 V, then return to stable in 1 ms. Compared with Figure 6a, the voltage decline is 77% larger and response time is 41% longer. So the system with proposed algorithm shows much better performance in terms of load changing. 

(3) Experiments with line voltage changing condition

**Figure 8:**
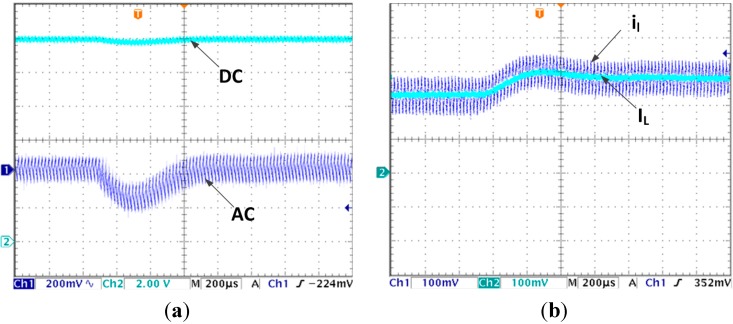
(**a**) Output voltage transient response due to input voltage changes from 6 V to 5 V with proposed algorithm; (**b**) Inductor current transient response due to input voltage changes from 6 V to 5 V with proposed algorithm.

When the line voltage changes from 6 V to 5 V, the output voltage and inductor currents transient response waveforms are shown in Figure 8a,b, respectively. From Figure 8a, the output voltage decreases to 11.81 V and returns to stable state (12 V) in 680 µs. In Figure 8b, both the estimated current and actual current converge to stable in 680 µs. In addition the estimated control equals to the actual average value.

**Figure 9:**
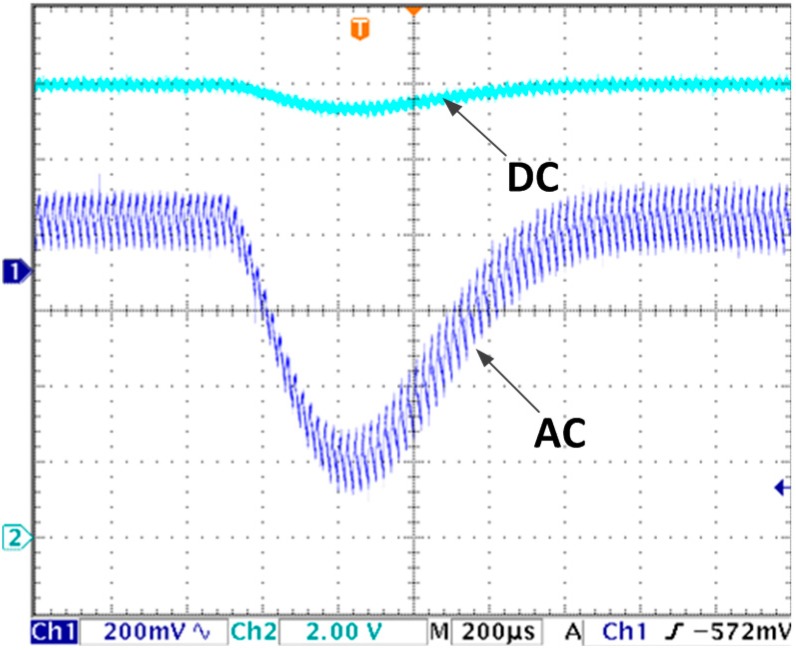
Output voltage transient response due to input voltage changes from 6 V to 5 V with voltage control mode.

For conventional voltage control mode, when the line voltages steps down from 6 V to 5 V. The output voltage waveform is shown in Figure 9. It decreases to 11.36 V first, then returns to 12 V in 1 ms time. Compared with Figure 8a, the response time is 47% longer and voltage drop is 237% larger.

From the above experimental results, the system presents very good robustness in both load and line voltage variation conditions by using proposed algorithm. Its transient response speed is much higher and its voltage drop is much less than the system with conventional voltage control mode. 

## 6. Conclusions

In this paper, a precise boost converter mathematical model, which includes a number of parasitic parameters, is built. The current estimation for boost converter sensorless control is realized by using an EKF current observer together with a LVEE module. The detailed analysis for LVEE module is carried out and the reason why it has the ability to eliminate output voltage steady state error can be clarified. For current control, the average current mode based PCC is applied. The current can reach its reference in two switching cycles. With all the above approaches, the system shows good performance in current estimation and dynamic response. In addition, the output voltage steady state error is also eliminated in load variation conditions by using LVEE module. These claims are all verified by experimental results.
